# Changes in plasma lipid composition upon glucocorticoid treatment in patients with primary immune thrombocytopenia

**DOI:** 10.1002/ctm2.70321

**Published:** 2025-05-07

**Authors:** Lili Ji, Yanxia Zhan, Shanshan Qin, Jianjun Jin, Mengjia Qian, Bijun Zhu, Yang Ou, Pengcheng Xu, Xia Shao, Hao Chen, Yunfeng Cheng

**Affiliations:** ^1^ Department of Hematology Zhongshan Hospital Fudan University Shanghai China; ^2^ Institute of Clinical Science Zhongshan Hospital Fudan University Shanghai China; ^3^ Center for Tumor Diagnosis & Therapy Jinshan Hospital Fudan University Shanghai China; ^4^ Department of Thoracic Surgery Zhongshan‐Xuhui Hospital Fudan University Shanghai China; ^5^ Department of Hematology Qingpu Branch Zhongshan Hospital Fudan University Shanghai China

1

Dear Editor,

Approximately one‐third of patients with primary immune thrombocytopenia (ITP) failed to glucocorticoid treatment and currently no biomarker can predict the response.^1^, ^2^ Lipidomics, a branch of metabolomics, offers a powerful approach to elucidate disease mechanisms and to discover disease‐specific biomarkers for diagnosis or therapy.^3^, ^4^ Here, we investigate the lipid portraits of ITP, in particular, illustrate the lipid metabolic characteristics and analyse the changes of lipids in ITP patients before and after glucocorticoid treatment. The study also sought to determine specific lipid species that could allow response prediction to glucocorticoid treatment.

The study was approved (approval no. B2020‐279R) by the Ethics Committee of Zhongshan Hospital, Fudan University. First, to investigate the lipid portraits of ITP, we used an internal standards kit containing nine‐lipid subclass (5040156, SCIEX) and the AB SCIEX QTRAP 5500 system to measure plasma lipids of 53 patients with ITP (newly diagnosed without treatment) and 20 healthy controls (HCs) matched by gender and age. Characteristics and clinical data of the patients and HC are included in Table S1. Recent studies have revealed the association of low high‐density lipoprotein cholesterol (HDL‐C) with high risk of autoimmune diseases, including ITP.^5^ The level of HDL‐C in the study showed significantly decreased in ITP (*p *= .001), similar to previous studies.^6^

Signalling lipids control multiple important cellular processes through signal transduction. Abnormality of lipid metabolism is considered a risk factor in many diseases. In our study, a total of 783 lipids was detected in plasma samples and 570 lipids were observed in quality control samples with relative standard deviation less than 30%. Partial least squares discrimination analysis model showed no very clear separation between ITP and HC (Figure 1A,B), indicating that ITP does not globally alter lipid composition. Analysis of the nine lipid subclasses, we found that cholesteryl ester, triacylglycerol (TAG), sphingomyelin (SM), phosphatidylcholine (PC), diacylglycerol (DAG), lysophosphatidylcholine (LPC) and phosphatidylethanolamine (PE) have no significant differences (Figure 1C). However, ITP patients showed an increase in ceramide (CER, *p *= .033) and lysophosphatidylethanolamine (LPE, *p *= .026). CER is an important bioactive lipid and involved in a variety of important cellular processes.^7^ The increased CER in ITP patients indicated that activation of inflammatory signalling could increase ITP CER biosynthesis, consistent with previously report. The increased CER could further affect cells apoptosis, autophagy and proliferation, leading to immune disorders in ITP patients. Several studies demonstrated that LPE are involved in the cell signalling process, acts as an activator of neurotrophic, increases the level of intracellular calcium and stimulates chemotactic migration.^8^ Dysregulation of lipid signalling may contribute to inflammation and inappropriate leukocyte activation in autoimmune disorders. Interestingly, the absolute neutrophil count showed a significant increase in ITP patients in current study. The difference of lipid distribution in the group of ITP patients and HC is shown in Figure 1D. The distributions of carbon and bond for TAG, CER and LPE in groups are shown in Figure 1E,F.

**FIGURE 1:**
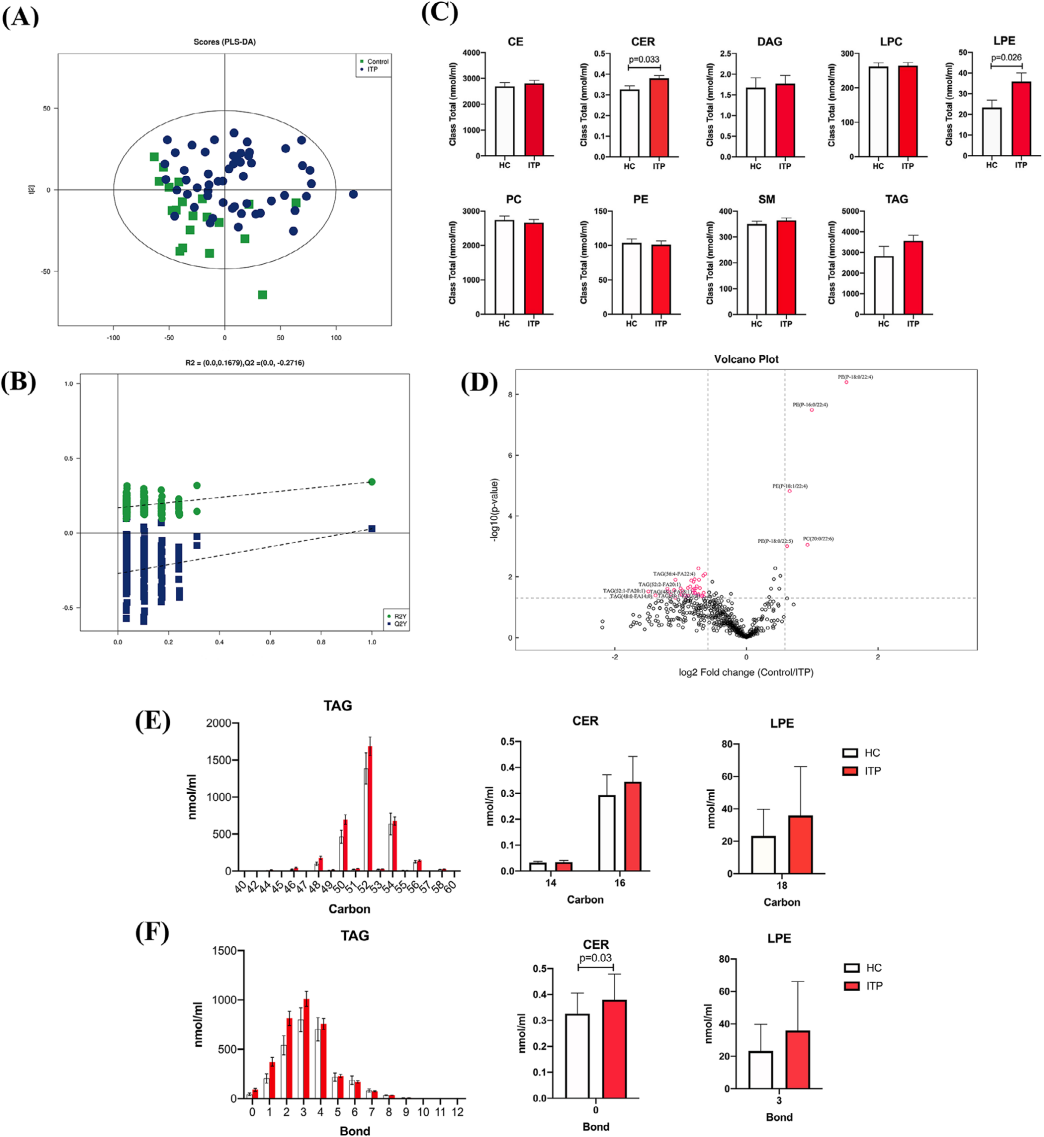
Lipid profile in patients with newly diagnosed primary immune thrombocytopenia (ITP). (A) Partial least squares discrimination analysis (PLS‐DA) score plot of ITP and healthy controls (HCs). (B) Validation plot of the PLS‐DA model. (C) Lipid subclasses from comparing ITP and HC. (D) Volcano plot showed the differential lipids between ITP and HC. Left pink indicated upregulated lipid species. Right pink indicated down‐regulated lipid species. Number of carbon (E) and bond (F) for triacylglycerol (TAG), ceramide (CER) and lysophosphatidylethanolamine (LPE). Each bar graph represents mean ± SEM. *p*‐Values less than.05 were labelled in the figure or indicated by asterisk ‘*’. CE, cholesteryl ester; DAG, diacylglycerol; LPC, lysophosphatidylcholine; PC, phosphatidylcholine; PE, phosphatidylethanolamine; SM, sphingomyelin.

We then assessed the changes of lipids in patients with ITP before and after treatment. Post‐treatment peripheral blood was obtained from patient 2 weeks after treatment. All of the enrolled patients received glucocorticoid treatment for extremely low platelet count and/or clinically significant bleeding, among which eight patients received high‐dose dexamethasone and 45 patients received prednisone or prednisolone. After the treatment, 28 were complete response (CR) and 25 were no response (NR) (the clinical characteristics of each patient are shown in Table S2).^9^ CER, LPC, LPE, PE, SM and TAG (Figure 2) were increased significantly in CR group than patients before treatment (*p *< .05). CER and LPE were also increased significantly in NR, compared to patients before treatment (*p *= .032 and.008, respectively). Intriguingly, however, as compared to patients before treatment, DAG was significantly decreased in NR (Figure 2C). Compared with CR, CER and PE were significantly decreased in NR (*p *< .05). Differences in the lipid profiles among different ITP states contribute to a better understanding of ITP lipid metabolism. The distributions of carbon and bond of lipids among ITP groups are shown in Figure S1. These results highlighted the complexity of lipidomics for ITP, and revealed potential associations between the altered lipid metabolism and ITP states.

**FIGURE 2:**
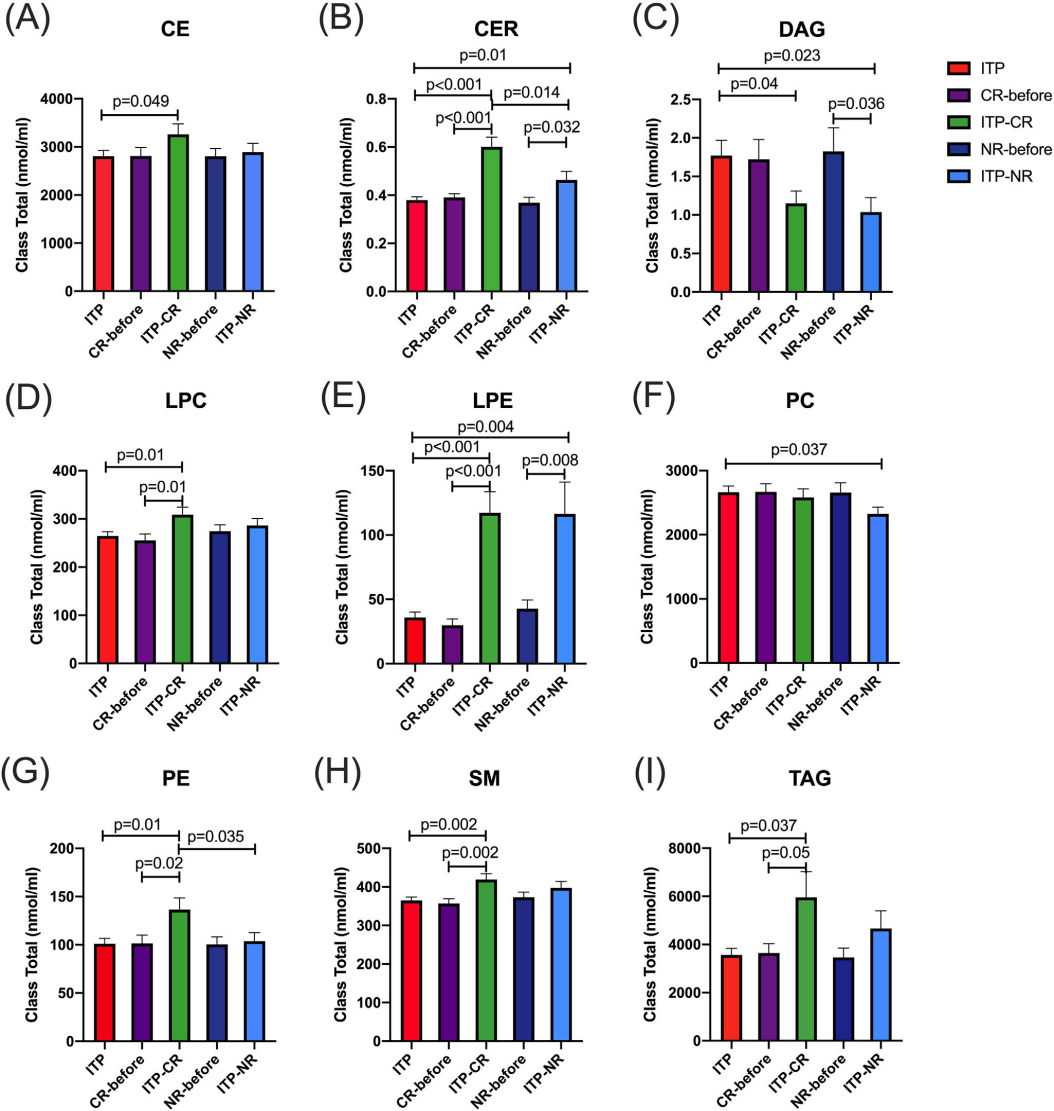
Glucocorticoids change the lipid profile in patients with primary immune thrombocytopenia (ITP). (A‒I) Lipid subclasses among ITP groups. Each bar graph represents mean ± SEM. *p*‐Values less than.05 were labelled in the figure. CE, cholesteryl ester; CER, ceramide; CR, complete response; DAG, diacylglycerol; LPC, lysophosphatidylcholine; LPE, lysophosphatidylethanolamine; NR, no response; PC, phosphatidylcholine; PE, phosphatidylethanolamine; SM, sphingomyelin; TAG, triacylglycerol.

Next, we explored the changes in lipid molecules that could impact disease without making the entire lipid class change. A total of 15 lipid species had significant different variable importance in projection (VIP > 1 and *p *< .05) in plasma levels between ITP patients and HC (Figure 3A). Thirty‐four lipid species had significant different between CR and ITP patients (Figure 3B), and 16 lipid species had significant different between NR and ITP patients (Figure 3C). These results suggested that the vast majorities of lipid composition have not changed in ITP, although significant alterations were observed in a number of lipid species. Lipid metabolomic studies have been diverse, some focused on different lipid classes, some used different detection methods or enrolled different patient groups. Of particular interest is the finding that PC (18:1/18:2) was significantly different between NR‐before and CR‐before group, indicating that PC (18:1/18:2) could be a potential biomarker to predict the response to glucocorticoid treatment (*p *= .019, VIP = 3.01) (Figure 4A). In addition, the percentage of PC (18:1/18:2) in total PC was increased significantly in NR‐before than CR‐before and HC (*p* = .002 and.046, respectively) (Figure 4B). The receiver operating characteristics (ROC) curve for PC (18:1/18:2) to discriminate glucocorticoid treatment response showed the area under the ROC curve (AUC) was.669 (95% confidence interval:.522‒.815, *p *= .036) (Figure 4C). However, the finding of the present study has limitations, small size of samples, in particular, the PC (18:1/18:2)’s AUC value of.669 to discriminate glucocorticoid treatment response require more samples to confirm its prediction power, and the direct relationship of lipid metabolism to immune function of ITP. As such, further large scale and further mechanism research are warranted.

**FIGURE 3:**
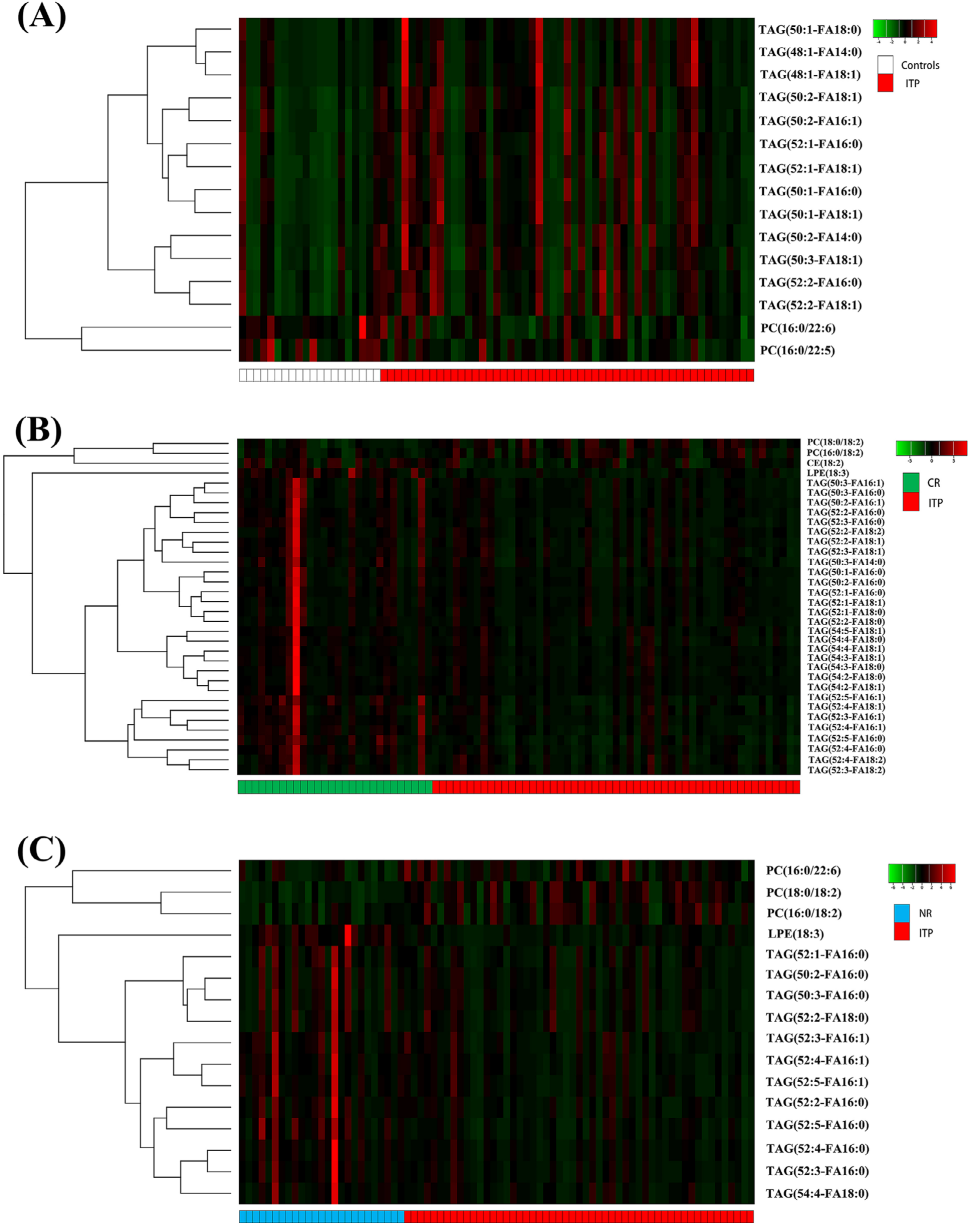
Changes of lipid species in primary immune thrombocytopenia (ITP) patients. Heatmap for individual lipids (VIP > 1 and *p *< .05) between groups for ITP and healthy controls (A), complete response (CR) and ITP (B), and no response (NR) and ITP (C). CE, cholesteryl ester; LPE, lysophosphatidylethanolamine; PC, phosphatidylcholine; TAG, triacylglycerol.

**FIGURE 4:**
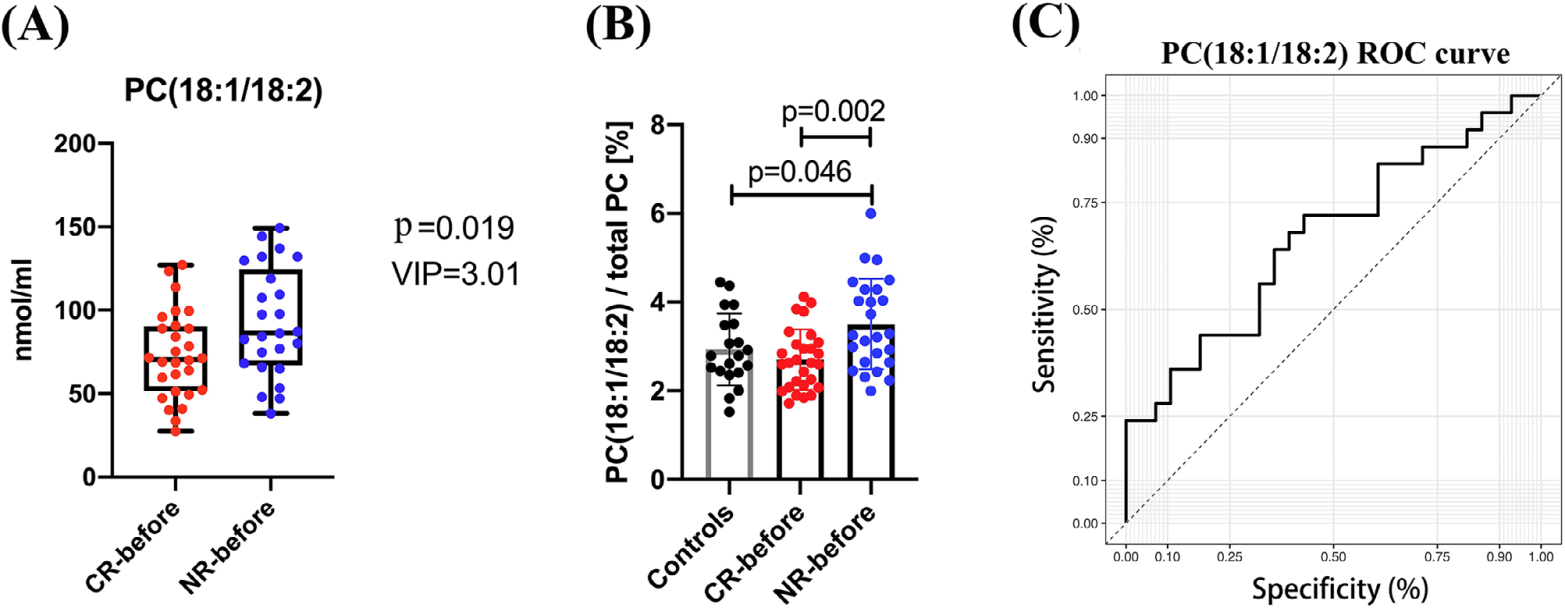
Change of phosphatidylcholine (PC) (18:1/18:2) in patients with primary immune thrombocytopenia (ITP). (A) Plasma level of PC (18:1/18:2) between complete response (CR)‐before and no response (NR)‐before. VIP and *p‐*values were labelled in the figure. (B) Percentage of PC (18:1/18:2) in total PC in healthy controls, CR‐before, and NR‐before. (C) Receiver operating characteristics (ROC) curves for PC (18:1/18:2) in distinguishing NR‐before from CR‐before. Area under the ROC curve (AUC) for determining NR to glucocorticoid treatment was.669 (*p *= .036, 95% confidence interval [CI]:.522‒.815). *p*‐Values less than.05 were labelled in the figure.

Lipidomics creates a powerful way to study lipid‐associated mechanisms in ITP and develops a new category of disease‐specific biomarkers and therapeutic targets. The study revealed that lipid metabolism plays a crucial role in ITP. PC (18:1/18:2) could be a potential biomarker to predict the efficacy of glucocorticoid treatment.

## AUTHOR CONTRIBUTIONS

Lili Ji, Yanxia Zhan, Hao Chen and Yunfeng Cheng conceived the study. Lili Ji, Yanxia Zhan, Hao Chen and Yunfeng Cheng performed the literature review and drafted and revised the manuscript. Hao Chen and Yunfeng Cheng contributed to the critical revision of the manuscript. Shanshan Qin, Yanxia Zhan, Jianjun Jin, Mengjia Qian, Bijun Zhu, Lili Ji, Pengcheng Xu, Xia Shao and Yang Ou performed the experiments and analysed the data. All authors read and approved the final manuscript.

## CONFLICT OF INTEREST STATEMENT

The authors declare that there is no conflicts of interest regarding the publication of this paper.

## ETHICS STATEMENT

The study was in accordance with the ethical standards formulated in the Helsinki Declaration and was approved by the Ethics Committee of Zhongshan Hospital, Fudan University (approval no. B2020‐279R). Written informed consent was obtained from each participant included in the study.

## Supporting information




**FIGURE S1** Distributions of carbon and bond for lipids in primary immune thrombocytopenia (ITP) patients. Number of carbon (A) and bond (B) for triacylglycerol (TAG), ceramide (CER), lysophosphatidylethanolamine (LPE) and sphingomyelin (SM) among ITP groups. Each bar graph represents mean ± SEM. CR, complete response; NR, no response.

Supporting Information

## Data Availability

All supporting data are included within the main manuscript and its supporting information. The original data used to support the findings of this study are available from the corresponding author upon request.
