# Supplementary figures and images for: Changes in plasma lipid composition upon glucocorticoid treatment in patients with primary immune thrombocytopenia
Source: Clin Transl Med. 2025 May 7;15(5):e70321. doi: 10.1002/ctm2.70321 (PMC12059205; doi:10.1002/ctm2.70321)

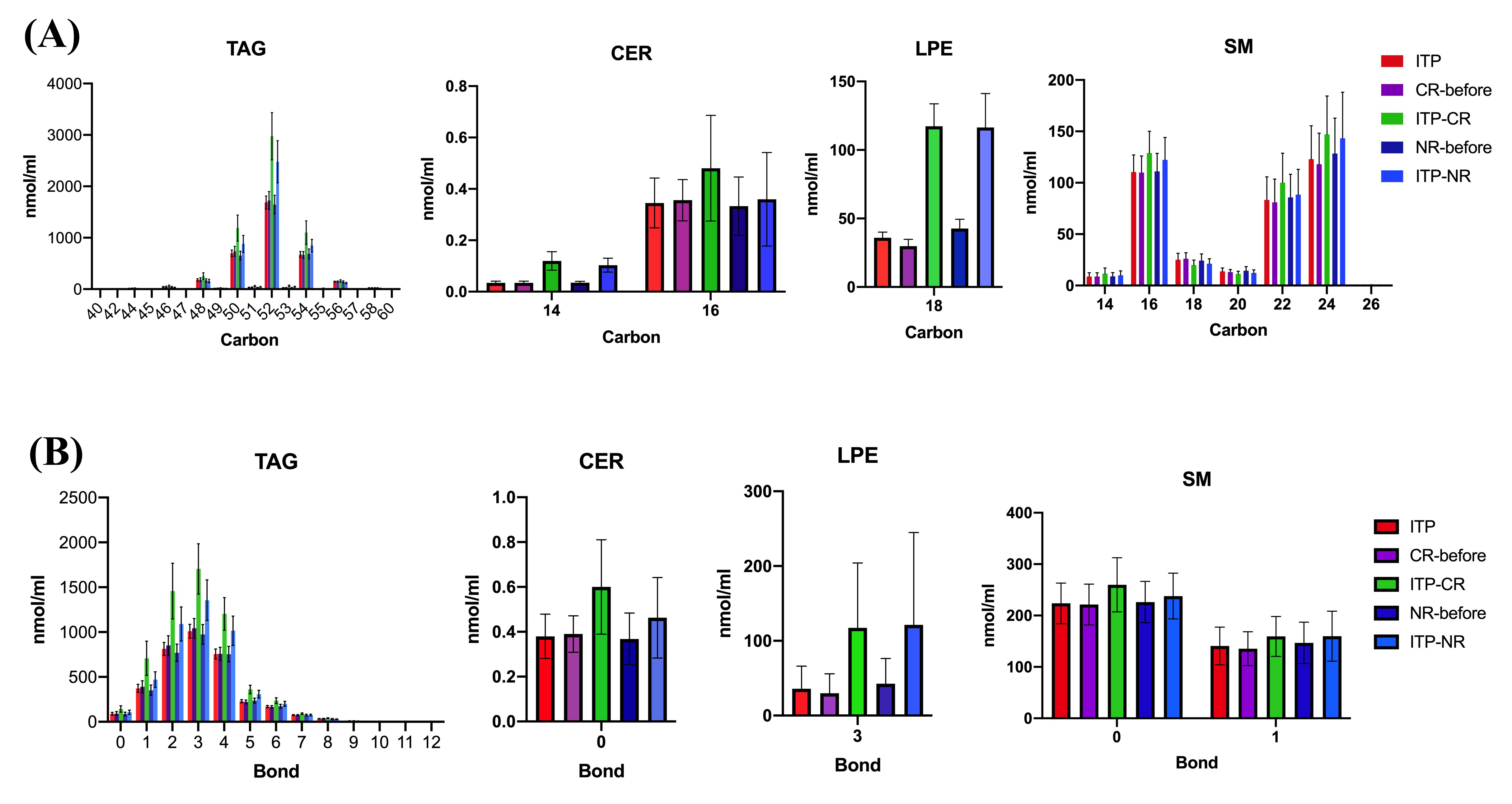

Supplement: Supplementary file 1 — FIGURE S1 Distributions of carbon and bond for lipids in primary immune thrombocytopenia (ITP) patients. Number of carbon (A) and bond (B) for triacylglycerol (TAG), ceramide (CER), lysophosphatidylethanolamine (LPE) and sphingomyelin (SM) among ITP groups. Each bar graph represents mean ± SEM. CR, complete response; NR, no response. [file CTM2-15-e70321-s001.jpg]
