# Supplementary material for: Changes in plasma lipid composition upon glucocorticoid treatment in patients with primary immune thrombocytopenia
Source: Clin Transl Med. 2025 May 7;15(5):e70321. doi: 10.1002/ctm2.70321 (PMC12059205; doi:10.1002/ctm2.70321)
Supplement: Supplementary file 2 — Supporting Information [file CTM2-15-e70321-s002.docx]

**Supplementary tables**

**TABLE S1** Demographics, routine hematological parameters, and bleeding scores on study participants.

| Variables | ITP | | | | HC | *P* ^b^ |
| --- | --- | --- | --- | --- | --- | --- |
|  | Total | Pre-CR | Pre-NR | | (*n*=20) |  |
|  | *n*=53 | *n*=28 | *n*=25 | |  |  |
| Age ^a^  (range) | 49.3 ± 14.2  (19-70) | 50.6 ± 15.1  (19-70) | 47.9 ± 13.4  (21-67) | | 43.2 ± 11.7  (26-66) | NS |
| Sex |  |  |  | |  | NS |
| Female | 28 | 12 | 16 | | 13 |  |
| Male | 25 | 16 | 9 | | 7 |  |
| Platelet count (×10^9^/L), median (range) | 11 (1-28) | 7(1-28) | 11(1-28) | | 228 (173-336) | <0.0001 |
| Total cholesterol (TC: mmol/L) ^a^ | 4.32 ± 1.21 | 4.2 ± 0.98 | 4.5 ± 1.4 | | 4.59 ± 0.53 | NS |
| Triglyceride (TG: mmol/L) ^a^ | 1.3 ± 0.69 | 1.2 ± 0.6 | 1.4 ± 0.8 | | 1.19 ± 0.54 | NS |
| Low-density lipoprotein cholesterol (LDL-C: mmol/L) ^a^ | 2.57 ± 0.88 | 2.5 ± 0.82 | 2.7 ± 0.9 | | 2.54 ± 0.59 | NS |
| High-density lipoprotein cholesterol (HDL-C: mmol/L) ^a^ | 1.15 ± 0.39 | 1.1 ± 0.4 | 1.2 ± 0.4 | | 1.51 ± 0.44 | 0.001 |
| Absolute neutrophil count (×10^9^/L) ^a^ | 5.63 ± 2.65 | 5.6 ± 2.3 | 5.6 ± 3.0 | | 3.34 ± 0.94 | 0.0003 |
| Bleeding score at initial diagnosis | 2.34 (1-5) | 2.5 (1-5) | 2.2 (1-5) | |  |  |
| mean (range) |  |  | |  |  |  |

^a^, data are presented as mean ± SD; ^b^, The *P* value is the comparison between ITP (Total) and HC. CR, complete response; NR, no response; ITP, primary immune thrombocytopenia; HC healthy controls.

**TABLE S2** Clinical characteristics of ITP patients.

| Patient number | Sex | Age | Platelet counts (×10^9^/L) | | Bleeding score | Platelet antibody | BMI  (kg/m^2^) |
| --- | --- | --- | --- | --- | --- | --- | --- |
|  |  |  | Before treatment | After treatment |  |  |  |
| 1 | M | 59 | 28 | 140 | 2 | Pos | 25 |
| 2 | F | 27 | 4 | 219 | 1 | Neg | 21.2 |
| 3 | M | 68 | 1 | 273 | 2 | Neg | 28 |
| 4 | M | 40 | 28 | 143 | 1 | Pos | 23.5 |
| 5 | F | 46 | 1 | 226 | 2 | Neg | 24.3 |
| 6 | M | 60 | 1 | 298 | 1 | Neg | 26.8 |
| 7 | M | 68 | 9 | 164 | 3 | Neg | 24.6 |
| 8 | M | 32 | 12 | 224 | 2 | Pos | 20.9 |
| 9 | M | 43 | 17 | 350 | 2 | Pos | 23.3 |
| 10 | F | 55 | 1 | 237 | 3 | Pos | 29 |
| 11 | F | 35 | 19 | 169 | 2 | Pos | 17.5 |
| 12 | M | 33 | 1 | 310 | 2 | Neg | 19.3 |
| 13 | M | 68 | 1 | 170 | 4 | Pos | 21.2 |
| 14 | F | 52 | 7 | 219 | 1 | Pos | 21.4 |
| 15 | M | 59 | 1 | 129 | 5 | Neg | 27.3 |
| 16 | F | 33 | 7 | 181 | 5 | Neg | 21.6 |
| 17 | F | 62 | 1 | 198 | 5 | Pos | 23.5 |
| 18 | F | 70 | 11 | 181 | 3 | Pos | 22.1 |
| 19 | F | 54 | 8 | 162 | 1 | Neg | 27.6 |
| 20 | M | 64 | 23 | 175 | 1 | Pos | 21 |
| 21 | M | 53 | 2 | 123 | 5 | Pos | 22.3 |
| 22 | F | 37 | 19 | 290 | 1 | Pos | 21.5 |
| 23 | M | 28 | 22 | 116 | 1 | Pos | 28 |
| 24 | M | 64 | 18 | 116 | 3 | Neg | 23.6 |
| 25 | M | 19 | 15 | 119 | 2 | Neg | 20.4 |
| 26 | M | 59 | 22 | 128 | 1 | Pos | 26.3 |
| 27 | F | 68 | 1 | 306 | 3 | Pos | 21 |
| 28 | F | 61 | 1 | 233 | 5 | Neg | 28.2 |
| 29 | M | 62 | 15 | 31 | 1 | Neg | 20.3 |
| 30 | F | 42 | 14 | 18 | 3 | Neg | 20.6 |
| 31 | M | 57 | 3 | 18 | 2 | Neg | 23.1 |
| 32 | M | 31 | 11 | 2 | 1 | Neg | 20.8 |
| 33 | M | 48 | 6 | 14 | 1 | Pos | 25 |
| 34 | F | 32 | 3 | 3 | 1 | Pos | 19.3 |
| 35 | F | 60 | 16 | 12 | 5 | Pos | 24 |
| 36 | F | 31 | 16 | 13 | 1 | Pos | 22.3 |
| 37 | F | 66 | 13 | 11 | 1 | Neg | 29 |
| 38 | F | 21 | 4 | 4 | 2 | Pos | 19.2 |
| 39 | M | 52 | 24 | 8 | 2 | Pos | 26 |
| 40 | F | 53 | 1 | 4 | 2 | Pos | 25.6 |
| 41 | M | 55 | 12 | 13 | 5 | Pos | 24 |
| 42 | F | 51 | 11 | 17 | 2 | Neg | 25.6 |
| 43 | M | 67 | 2 | 9 | 2 | Pos | 23 |
| 44 | F | 65 | 6 | 21 | 4 | Neg | 21.8 |
| 45 | M | 42 | 19 | 17 | 1 | Pos | 24.2 |
| 46 | F | 51 | 16 | 28 | 1 | Neg | 26.7 |
| 47 | F | 47 | 6 | 13 | 4 | Neg | 24.1 |
| 48 | F | 48 | 15 | 11 | 1 | Pos | 20.4 |
| 49 | F | 27 | 10 | 10 | 5 | Pos | 21.3 |
| 50 | F | 53 | 16 | 30 | 1 | Pos | 26.7 |
| 51 | M | 29 | 11 | 13 | 1 | Pos | 21 |
| 52 | F | 43 | 13 | 8 | 3 | Neg | 23.5 |
| 53 | F | 65 | 5 | 21 | 4 | Pos | 24.1 |

ITP, primary immune thrombocytopenia; Pos, positive; Neg, negative; BMI, body mass index.
